# Cell competition between wild-type and JAK2V617F mutant cells prevents disease relapse after stem cell transplantation in a murine model of myeloproliferative neoplasm

**DOI:** 10.1186/s40164-021-00241-2

**Published:** 2021-10-19

**Authors:** Haotian Zhang, Melissa Castiglione, Lei Zheng, Huichun Zhan

**Affiliations:** 1 Graduate Program in Molecular and Cellular Biology, Stony Brook University, Stony Brook, NY USA; 2 Department of Medicine, Stony Brook School of Medicine, Stony Brook, NY USA; 3 Albert Einstein College of Medicine, Bronx, NY USA; 4 The Sidney Kimmel Comprehensive Cancer Center, Johns Hopkins University, Baltimore, MD USA; 5 Medical Service, Northport VA Medical Center, Northport, NY USA; 6 Division of Hematology-Oncology, Department of Medicine, Stony Brook School of Medicine, HSC T15, Room 053, Stony Brook, NY 11794 USA; 7 Northport VA Medical Center, Building 62, Room 124, 79 Middleville Road, Northport, NY 11768 USA

**Keywords:** Myeloproliferative neoplasm, Stem cell transplantation, Relapse, Cell competition, JAK2V617F, Immune cells, PD-L1, Murine model

## Abstract

Disease relapse after allogeneic stem cell transplantation is a major cause of treatment-related morbidity and mortality in patients with myeloproliferative neoplasms (MPNs). The cellular and molecular mechanisms for MPN relapse are not well understood. Here, we established a murine model of MPN relapse, in which ~ 60% of the MPN recipient mice develop disease relapse after receiving stem cell transplantation with wild-type marrow donor. Using this model, we find that impaired wild-type cell function is associated with MPN disease relapse. We also show that competition between wild-type and JAK2V617F mutant cells can modulate the immune cell composition and PD-L1 expression induced by the JAK2V617F oncogene. These results suggest that cell competition between wild-type donor cells and JAK2V617F mutant recipient cells can prevent MPN disease relapse after stem cell transplantation.

**Supplementary Information:**

The online version contains supplementary material available at 10.1186/s40164-021-00241-2.

To the Editor.

Allogeneic stem cell transplantation is the only curative treatment for patients with myeloproliferative neoplasms (MPNs). However, disease relapse is seen in up to 40% of patients after transplantation and is a leading cause of transplant-related morbidity and mortality in these patients [1–4]. Mechanisms for why the MPN disease relapses in some patients while remains in remission in others are not well understood. The hematopoietic stem/progenitor cell (HSPC) compartment in MPN is heterogeneous with the presence of both JAK2 wild-type and JAK2V617F mutant cells in most patients [5]. Recently, we reported that co-existing wild type cells can alter both the gene expression profile and cellular function of JAK2V617F mutant HSPCs and prevent the expansion of mutant cells [6]. We hypothesize that competition between the wild-type donor and JAK2V617F mutant recipient cells dictates the outcome of disease relapse versus remission after stem cell transplantation.

To test this hypothesis, we crossed JAK2V617F Flip-Flop (FF1) mice with Tie2-Cre mice to express JAK2V617F specifically in all hematopoietic cells and vascular endothelial cells (ECs) (Tie2^±^FF1^±^, or Tie2FF1) [7–9], so as to model the human diseases in which both the HSPCs and ECs harbor the mutation [10–12]. We transplanted wild-type CD45.1 marrow directly into lethally irradiated Tie2FF1 mice or Tie2-cre control mice (CD45.2) (Fig. 1A). During a ~ 6-7mo follow up, while all wild-type control recipients displayed full donor engraftment, ~ 60% Tie2FF1 recipient mice displayed recovery of the JAK2V617F mutant hematopoiesis (mixed donor/recipient chimerism) 10 weeks after transplantation and developed neutrophilia and thrombocytosis, results consistent with our previous report [8] (Fig. 1B, C). Marrow Lin^−^cKit^+^Sca1^+^CD150^+^CD48^−^ HSCs were significantly expanded in the Tie2FF1 recipient mice with mixed chimerism (i.e., with disease relapse) compared to Tie2FF1 recipient mice with full donor engraftment (i.e., with disease remission) (Fig. 1D).

**Fig. 1:**
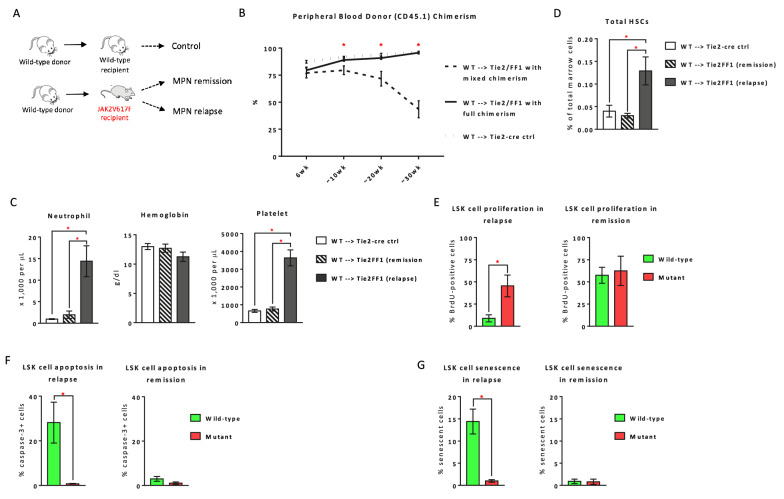
MPN disease relapse in a JAK2V617F-bearing vascular niche following lethal irradiation and marrow transplantation. **A** A murine model of MPN disease relapse established by marrow transplantations. **B** Peripheral blood CD45.1 chimerism following transplantation of wild-type (WT) CD45.1 marrow cells into lethally irradiated Tie2FF1 mice or Tie2-cre control mice (CD45.2) (n  =  8–12 mice in each group). **C** Tie2FF1 recipients with mixed chimerism developed both neutrophilia and thrombocytosis (n  =  5–8 mice in each group). **D** Total marrow Lin^−^cKit^+^Sca1^+^CD150^+^CD48^−^ HSCs (n  =  4–9 mice in each group). **E** Lin^−^cKit^+^Sca1^+^ (LSK) cell proliferation rate in relapse (left) and remission (right) Tie2FF1 recipient mice, measured by in vivo BrdU labeling (left: n  =  6 mice in each group; right: n  =  4 mice in each group). **F** Cellular apoptosis rate of wild-type and JAK2V617F mutant LSKs in relapse (left) and remission (right) Tie2FF1 recipient mice, measured by activated caspase-3 staining using flow cytometry analysis (left: n  =  4–6 mice in each group; right: n  =  3–5 mice in each group). **G** Cellular senescence rate of wild-type and JAK2V617F mutant LSKs in relapse (left) and remission (right) Tie2FF1 recipient mice, measured by SA-b-Gal activity using flow cytometry analysis (left: n  =  6 mice in each group; right: n  =  4–5 mice in each group). *P  <  0.05

We compared wild-type and JAK2V617F mutant HSPC functions between the relapsed Tie2FF1 recipient mice and those remained in remission. We found that wild-type Lin^−^cKit^+^Sca1^+^ (LSK) HSPCs demonstrated decreased proliferation, increased apoptosis, and increased cellular senescence compared to mutant HSPCs during disease relapsed; in contrast, there was no significant difference between wild-type and mutant HSPC functions (i.e., proliferation, apoptosis, senescence) during remission (Fig. 1E–G). These findings suggest that deterioration of wild-type cell function is associated with MPN disease relapse.

To understand how co-existing wild-type cells prevent the expansion of JAK2V617F mutant HSPCs, we used a murine model of wild-type and JAK2V617F mutant cell competition we previously established [6]. In this model, when 100% JAK2V617F mutant marrow cells are transplanted alone into lethally irradiated wild-type recipients, the recipient mice develop a MPN phenotype ~ 4wks after transplantation; in contrast, when a 50–50 mix of mutant and wild-type marrow cells are transplanted together into lethally irradiated wild-type recipient mice, the mutant donor cells engraft to a similar level as the wild-type donor cells and the recipient mice display normal blood counts during more than 4-months of follow up [6] (Fig. 2A). Gene expression profiling revealed that gene ontology terms humoral immune response, leukocyte/B cell/T cell/complement activation, and immune response-activated signaling transduction were highly enriched in JAK2V617F mutant Lin^−^cKit^+^ HSPCs with cell competition compared to mutant HSPCs without competition (Fig. 2B). These results prompted us to examine various immune cell types and we found that: (1) compared to wild-type HSPCs, JAK2V617F mutant HSPCs generated significantly more T cells and less B cells in the spleen, and more myeloid-derived suppressor cells (MDSCs) in the marrow; and (2) there was no difference in T, B, or MDSC numbers between recipients of wild-type HSPCs and recipients of mixed wild-type and JAK2V617F mutant HSPCs (Fig. 2C). Similarly, we found that program death ligand 1 (PD-L1) expression was significantly upregulated on JAK2V617F mutant LSK cells compared to wild-type LSKs; however, this PD-L1 upregulation on mutant LSKs was significantly decreased with co-existing wild-type cell competition (Fig. 2D). Taken together, these results indicate that the wild-type cells may prevent the expansion of co-existing JAK2V617F mutant cells through modulating the immune abnormality induced by the JAK2V617F mutation.

**Fig. 2:**
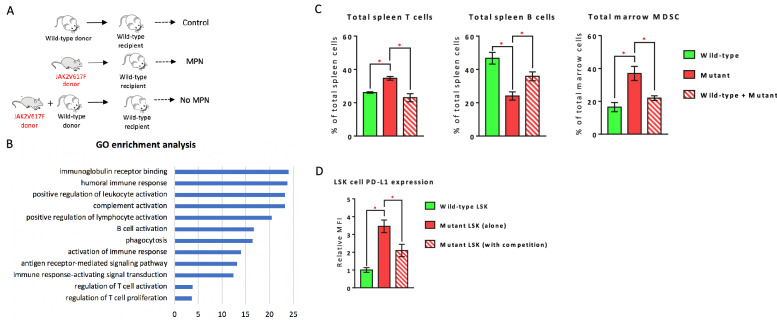
Immune regulation associated with wild-type and JAK2V617F mutant cell competition. **A** A murine model of wild-type and JAK2V617F mutant cell competition established by marrow transplantations. **B** Differentially enriched Gene Ontology (GO) terms in mutant Lin^−^cKit^+^ HSPCs transplanted together with wildtype cells (pooled sample from 3 mice) compared to mutant HSPCs transplanted alone (pooled sample from 2 mice). *P* values are plotted as the negative of their logarithm. **C** Spleen T cells (CD3^+^CD4^+^and CD3^+^CD8^+^) and B cells (CD3^−^B220^+^), and marrow MDSCs (both CD11b^+^Ly6ChighLy6G^−^ M-MDSCs and CD11b^+^Ly6ClowLy6G^+^PMN-MDSCs) in wild-type recipient of wild-type donor (“wild-type”), JAK2V617F mutant donor (“mutant”), or both wild-type and mutant donors (“wild-type + mutant”) (spleen T cells: n  =  3 mice in each group; spleen B cells: n  =  3–6 mice in each group; marrow MDSCs: n  =  3–6 mice in each group). **D** Quantitative measurement of mean fluorescence intensity for PD-L1 staining on wild-type LSK cells (n  =  7 mice), JAK2V617F mutant LSK cells (n  =  4 mice), and JAK2V617F mutant LSK cells with co-existing wild-type cell competition (n  =  9 mice). *P  <  0.05

In summary, although the molecular mechanisms responsible for MPN disease relapse after stem cell transplantation remain unclear, our study provides important observations and mechanistic insights that co-existing wild-type cell competition can prevent MPN disease relapse after stem cell transplantation. Findings from our study also suggest that one possible mechanism for cell competition to prevent MPN disease relapse is that wild-type cells can help restore the immune dysregulation induced by the JAK2V617F oncogene. Results from our previous work^6^ and current study provide the rational to further investigate whether wild-type cells could be used as a therapeutic approach to control mutant clonal expansion in MPNs. Additional methods can be found in Additional file 1.

## Supplementary Information

**Additional file 1.** Materials and methods.

## Data Availability

Upon request to the corresponding author.
