# Supplementary material for: Cell competition between wild-type and JAK2V617F mutant cells prevents disease relapse after stem cell transplantation in a murine model of myeloproliferative neoplasm
Source: Exp Hematol Oncol. 2021 Oct 19;10:47. doi: 10.1186/s40164-021-00241-2 (PMC8524893; doi:10.1186/s40164-021-00241-2)
Supplement: Supplementary file 1 — Additional file 1. Materials and methods. [file 40164_2021_241_MOESM1_ESM.pdf]

Materials and Methods

Experimental mice

JAK2V617F Flip-Flop (FF1) mice[18] was provided by Radek Skoda (University Hospital, Basal, Switzerland) and *Tie2-Cre* mice[19] by Mark Ginsberg (University of California, San Diego). FF1 mice were crossed with Tie2-Cre mice to express JAK2V617F specifically in all hematopoietic cells (including HSPCs) and vascular ECs ($Tie2^{+/-}$ $FF1^{+/-}$, or Tie2FF1), so as to model the human diseases in which both the hematopoietic stem cells and ECs harbor the mutation. All mice used were crossed onto a C57BL/6 background and bred in a pathogen-free mouse facility at Stony Brook University. Animal experiments were performed in accordance with the guidelines provided by the Institutional Animal Care and Use Committee.

Stem cell transplantation assays

Recipient mice were irradiated with two doses of 540 rad 3 hours apart. Donor cells were injected into recipients by standard intravenous tail vein injection using a 27G insulin syringe. For competitive transplantation, $5x10^5$ CD45.2 donor marrow cells from Tie2FF1 mice were injected intravenously together with $5x10^5$ competitor CD45.1 wild-type marrow cells. For noncompetitive transplantation, $1x10^6$ unfractionated donor marrow cells were transplanted into wild-type recipients by intravenous tail vein injection.

Complete blood counts

Peripheral blood was obtained from the facial vein via submandibular bleeding, collected in an EDTA tube, and analyzed using a HM5 Hematology Analyzer (Zoetis, Parsippany, NJ).

Marrow and spleen cell isolation

Murine femurs and tibias were first harvested and cleaned thoroughly. Marrow cells were flushed into PBS with 2% fetal bovine serum using a 25G needle and syringe. Remaining bones were crushed with a mortar and pestle followed by enzymatic digestion with DNase I (25U/ml) and Collagenase D (1mg/ml) at 37 °C for 20 min under gentle rocking. Tissue suspensions were thoroughly homogenized by gentle and repeated mixing using 10ml pipette to facilitate dissociation of cellular aggregates. Resulting cell suspensions were then filtered through a 40uM cell strainer.

Murine spleens were collected and placed into a 40uM cell strainer. The plunger end of a 1ml syringe was used to mash the spleen through the cell strainer into a collecting dish. 5ml PBS with 2% FBS was used to rinse the cell strainer and the resulting spleen cell suspension was passed through a 5ml syringe with a 23G needle several times to further eliminate small cell clumps.

Flow cytometry

All samples were analyzed by flow cytometry using a FACSAriaTM III or a LSR II (BD Biosciences, San Jose, CA, USA). CD45.1 (Clone A20, BD Biosciences), CD45.2 (Clone 104, Biolegend, San Diego, CA), Lineage cocktail (include CD3, B220, Gr1, CD11b, Ter119; Biolegend), cKit (Clone 2B8, Biolegend), Sca1 (Clone D7, Biolegend), CD150 (Clone mShad150, eBioscience, San Diego, CA), CD48 (Clone HM48-1, Biolegend), PD-L1 (Clone 10F.9G2, Biolegend), CD3 (Clone 17A2, Biolegend), CD4 (Clone GK1.5, Biolegend), CD8 (Clone 53-6.7, Biolegend), B220 (Clone RA3-6B2, Biolegend), CD11b (Clone M1/70, Biolegend), Ly-6C (Clone HK1.4, Biolegend), and Ly-6G (Clone 1A8, Biolegend) antibodies were used.

BrdU incorporation analysis

Mice were injected intraperitoneally with a single dose of 5-bromo-2′-deoxyuridine (BrdU; 100 mg/kg body weight) and maintained on 1mg BrdU/ml drinking water for two days. Mice were then euthanized and marrow cells isolated as described above. For analysis of HSPC ($Lin^-cKit^+Sca1^+$) proliferation, marrow cells were first stained with fluorescent antibodies specific for cell surface HSPC markers, followed by fixation and permeabilization using the Cytofix/Cytoperm kit (BD Biosciences), DNase digestion (Sigma, St. Louis, MO), and anti-BrdU antibody (Biolegend) staining to analyze BrdU incorporation[20]. Data were acquired using a LSR II flow cytometer.

Analysis of apoptosis by active caspase-3 staining

Marrow cells were stained with fluorescent antibodies specific for cell surface HSPC markers, followed by fixation and permeabilization using the Cytofix/Cytoperm kit (BD Biosciences). Cells were then stained using a rabbit anti-activated caspase-3 antibody[20]. Data were acquired using a LSR II flow cytometer.

Analysis of senescence by senescence associated β-galactosidase (SA-β-Gal) activity

Marrow cells were stained with fluorescent antibodies specific for cell surface HSPC markers. Cells were then washed and fixed using 2% paraformaldehyde and incubated with CellEvent^{TM} Senescence Green Probe (ThermoFisher Scientific, Waltham, MA) according to the manufacturer's instruction. Data were acquired using a LSR II flow cytometer.

Transcriptome analysis using RNA sequencing
For marrow HSPC RNA sequencing (RNA-seq), wild-type (CD45.1) and JAK2V617F mutant (CD45.2) marrow Lin^-cKit^+ HSPCs were isolated by magnetic bead isolation (with 90-95% purity) (Miltenyi Biotec, San Diego, CA). Briefly, the Lineage Cell Depletion Kit (Miltenyi Biotec) was used to deplete mature hematopoietic cells. The lineage negative cells were collected and then positively selected for $CD117^+$ ($cKit^+$) cells using CD117 microbead (Miltenyi Biotec) to yield Lin^-cKit^+ HSPCs. Messenger RNA samples of wild-type Lin-cKit+ HSPCs transplanted alone (one pooled sample from 3 mice), JAK2V617F mutant HSPCs transplanted together with wild-type cells (one pooled sample from 3 mice), and JAK2V617F mutant HSPCs transplanted alone (one pooled sample from 2 mice) were assessed by RNA-seq as we previously described[20]. Gene Ontology (GO) (http://www.geneontology.org/) enrichment analysis of differentially expressed genes was implemented by the ClusterProfiler R package. GO terms with corrected p values < 0.05 were considered significantly enriched.

Statistical Analysis
Statistical analyses were performed using Student's unpaired, 2-tailed *t* tests using Excel software (Microsoft). A p value of less than 0.05 was considered significant. Data are presented as mean ± standard error of the mean (SEM).
